# Perceptions of Family Support in the Stroke Prevention Program Make My Day

**DOI:** 10.1177/00084174261433649

**Published:** 2026-04-09

**Authors:** Kristin Norefors, Eric Asaba, Elisabet Åkesson, Lisette Farias, Ann-Helen Patomella

**Keywords:** Health prevention, Intervention, Lifestyle, Relatives, Social environment, Environnement social, famille, intervention, mode de vie, prévention en santé

## Abstract

**Background.** Numerous risk factors for stroke are modifiable and are associated with lifestyle and daily habits. An occupational perspective in prevention, which emphasizes habits and routines, can facilitate sustainable and healthy lifestyle changes. Additionally, lifestyle changes generally take place within a social context, making family involvement indispensable. **Purpose.** This study aims to explore family support from the perspective of persons with risk for stroke (PWRS) who participated in an occupation-based stroke prevention program, and their family members. **Method.** Explorative qualitative design using interviews was conducted with 10 PWRS who participated in the Make My Day intervention and 10 family members of intervention participants, including both married and nonmarried partners and adult children. Data were analyzed using an inductive semantic approach and reflexive thematic analysis. **Findings.** Family support depends on the nature of relationships, the family's willingness to accept support, and expectations. Three themes were generated through the analysis: (a) shared perspectives on lifestyle influence support, (b) balancing support and individual responsibility, and (c) generating tensions from different views on health and lifestyle habits. **Conclusion.** Family support encourages lifestyle changes by sharing activities, offering encouragement, and providing positive reinforcement. Nonetheless, difficulties can occur in maintaining a balance between supporting family members and respecting their personal responsibility, particularly when family members have varying levels of understanding regarding health.

## Résumé

**Description.** De nombreux facteurs de risque d’AVC sont modifiables et sont associés au mode de vie et aux habitudes quotidiennes. Une perspective occupationnelle en prévention, qui met l’accent sur les habitudes et les routines, peut faciliter des changements de mode de vie durables et sains. En outre, les changements de mode de vie se produisent généralement dans un contexte social, rendant la participation de la famille indispensable. **But.** Cette étude vise à explorer le soutien familial du point de vue des personnes présentant un risque d’AVC (PRAVC) qui ont participé à un programme de prévention de l’AVC fondé sur l’occupation, et de celui de membres de leur famille. **Méthodologie.** Une étude qualitative exploratoire basée sur des entretiens a été menée auprès de 10 PRAVC qui ont participé à l’intervention Make May Day et de 10 membres de leur famille, dont des conjoints (mariés et non mariés) et des enfants d’âge adulte. Les données ont été analysées à l’aide d’une approche sémantique inductive et d’une analyse thématique réflexive. **Résultats.** Le soutien de la famille dépend de la nature des relations, de la volonté de la famille d’accepter le soutien ainsi que des attentes. Trois thèmes ont été générés par l’analyse : (1) Des perspectives partagées sur le mode de vie influencent le soutien; (2) L’équilibre entre le soutien et la responsabilité individuelle; et (3) Les tensions générées par les différences de points de vue sur la santé et les habitudes de vie. **Conclusion.** Le soutien familial encourage les changements de mode de vie par le partage d’activités, l’offre d’encouragements et l’apport d’un renforcement positif. Néanmoins, il peut être difficile de maintenir un équilibre entre soutenir les membres de sa famille et respecter leur responsabilité personnelle, en particulier lorsque les membres de la famille ne comprennent pas tous la santé de la même façon.

## Background

Stroke is globally among the most common noncommunicable diseases (Feigin et al., 2021). Most risk factors for stroke are modifiable, and incorporating healthy lifestyle habits into everyday life can be associated with fewer stroke risk factors (Franklin et al., 2020; Goldstein et al., 2011). Secondary prevention of stroke should include family support to facilitate lifestyle changes (Lawrence et al., 2016). Family support includes informal help from family members (Kamaryati & Malathum, 2020). Lessons learned regarding family support and lifestyle changes may help enhance and refine future stroke prevention initiatives, including healthy habits and routines. Lifestyle Redesign^®^ studies focus on habits and routines through occupations embedded in everyday life and have reported positive outcomes in social participation, physical functioning, mental health, overall life satisfaction, and well-being among older adults (Barnes et al., 2008; Clark et al., 2012; Levasseur et al., 2019). In contexts outside North America, effects of similar programs focusing on lifestyle changes have varied (Johansson & Bjorklund, 2016; Ng et al., 2013). In one intervention drawing on Lifestyle Redesign^®^, including stroke survivors in Scandinavian settings, no significant differences were found in well-being, occupation, and social participation when compared to physical exercise interventions (Lund, Michelet, Kjeken et al., 2012a; Lund, Michelet, Sandvik et al., 2012b). Because different outcomes have been used and the angle of family perspectives has not been included in relation to illness prevention, there continue to be several knowledge gaps related to how principles of lifestyle change can be applied in occupational therapy interventions.

An occupational perspective on stroke prevention suggests that everyday activities influence health through habits, which can develop through personal experiences within sociocultural contexts (Fritz & Cutchin, 2017). Social support (i.e., family members) within the sociocultural context where people carry out their daily activities is recognized as a key factor in facilitating and maintaining lifestyle changes to reduce stroke risk factors in primary health care (PHC) (Mälstam et al., 2022; Murray et al., 2013). While the influence of family members in supporting lifestyle changes to lower stroke risk is limited, valuable insights can be gained from strategies used in other groups such as type 2 diabetes (Abel et al., 2018; Gupta et al., 2019) and weight management programs (Albright et al., 2020). Abel et al. (2018) highlighted the importance of supportive relationships during lifestyle change to reduce the risk of type 2 diabetes. Nonetheless, family support can manifest in various ways and may both facilitate and hinder lifestyle change (Abel et al., 2018; Gupta et al., 2019), and there remains a limited understanding of how family support influences lifestyle changes and their sustainability in the context of stroke prevention programs (Murray et al., 2013). More knowledge is needed about experiences and reasoning about family support in stroke prevention programs to guide the development and integration of family support in lifestyle change efforts.

## Objective

This study aimed to explore family support from the perspective of persons with risk for stroke (PWRS) who participated in an occupational-based stroke prevention program Make My Day (MMD) and their family members.

## Method

In alignment with social constructivism (Karnilowicz et al., 2014), an exploratory qualitative research design (Hunter et al., 2019) was used to examine the complexity of family support in the MMD prevention program and to explore PWRS and their families’ subjective experiences of support. This design allows participants to express their reflections in their own words, enhancing understanding of family support concepts in the MMD prevention program. Ethical approval was obtained from the Swedish Ethical Review Board in Stockholm, Sweden, Dnr: 2015/834-31, 2016/2203-32, 2019-01444, and 2021-05902-02.

## Settings/Context

This study was part of a larger research project that evaluates and explores the MMD intervention. The development of the MMD intervention follows the Medical Research Council (MRC) guidelines for complex interventions (Skivington et al., 2021). Participation in health-promoting, engaging occupations is central to the MMD program theory and runs like a red thread through the intervention themes. Core components of Michie et al. (2013) we used in the behavioral change strategy include capability, opportunity, and motivation from the COM-B model. In the COM-B model, family context is a key aspect of opportunity, as it represents external factors that can enable or hinder behavior change (Michie et al., 2011). The MMD prevention program draws on principles of Lifestyle Redesign^®^, such as a focus on daily activities and the implementation of relevant and sustainable activities applicable to lifestyle challenges (Clark et al., 1997, 2012). The MMD prevention program runs over 10 weeks, with six 90-min group sessions on different themes, for example, stroke risk factors, dietary habits, and physical activity, these themes are all related to engaging occupations for example physical activities that engage the person. MMD prevention uses individual activity analysis, and occupation-based goal setting, to support individuals with risk factors for stroke to make lifestyle changes and reduce risk factors for stroke. Information about the MMD prevention program can be found elsewhere (Patomella et al., 2023).

## Recruitment

Participants in the present study included two groups: (a) PWRS who have participated in the MMD program, and (b) family members of the PWRS. The PWRS and their family members were selected from a randomized controlled trial (Johnsson et al., 2025), and recruitment was conducted through social media by flyers at healthcare clinics in Region Stockholm at three time-points between 2022 and 2023. Inclusion criteria for the PWRS were: (a) age of 55–75 years; (b) three or more stroke risks factors, scored as high in the Stroke Risk Score Card; (c) two or more of the scored risk factors were modifiable (d); motivation to lifestyle change, by answering five questions and showing a moderate or high motivation for change (e) able and motivated to use a smartphone application (f) living in the Stockholm region. Inclusion criteria for family members were: (a) family member to PWRS; (b) a self-expressed interest in supporting the family member with a risk for stroke; and (c) ability to participate in interviews.

A total of 122 PWRS were included and divided into an intervention group (*n* = 63) and a control group (*n* = 59). Of the 63 participants in the intervention group, 49 attended through the 12-month follow-up and were invited to participate in interviews as part of the process evaluation. A total of 20 interviews were conducted with the PWRS. To select information-rich cases that include perspectives about family support while maintaining as balanced a gender distribution as possible within the PWRS group, purposive sampling was used. Of these 20 interviews with PWRS, 10 interviews were selected by the first author based on: (a) whether the PWRS has a family member (*n* = 16) and (b) the family members have been involved in the lifestyle change (*n* = 10). Family members were recruited using a convenience sampling strategy mediated by the PWRS in the intervention group (*n* = 63), as they were asked to identify a family member who would agree to participate in the research study. A researcher thereafter contacted these family members by email or phone, and, if they agreed to participate, they were invited to complete a survey on background information. Among the PWRS in the intervention group, 40 reported having a family member, and of these, 10 family members agreed to participate in an interview. Before data collection, PWRS in the MMD program and their family members received oral and written information about the study. All participants had the opportunity to ask questions about informed consent before committing.

## Data Gathering

Interviews with PWRS and their family members were conducted 12 months after the intervention, following the 12-month follow-up in the conducted RCT (Johnsson et al., 2025). The interviews with PWRS were conducted individually by research members EJ, CJ, and CE in an urban PHC or university setting. The interviews ranged from 34 to 72 min (median 48, ± 4.5 min). The first author, KN, and research member, CE, conducted interviews with individual family members. Interviews with family members were conducted by telephone and lasted between 13 and 28 min (median 20 ± 6 min). Interviews with PWRS and family members were semistructured, with digital voice recorders, and transcribed verbatim. PWRS interviews were transcribed by CJ and CE. Interviews with family members were transcribed by KN and students AA and IE.

The semistructured interview guide which was initially designed (Brinkmann & Kvale, 2015) and used to collect data for a pilot and feasibility study on MMD participation. The interview guide was later refined and adjusted as part of data collection for a process evaluation of the aforementioned RCT study (Johnsson et al., 2025). Interviews with the PWRS had open-ended questions about: (a) program content, (b) lifestyle changes, (c) family support, (d) stroke risk factors, and (e) engaging activities. Examples of questions in the interview guide were: Can you tell me about your current lifestyle and daily activity patterns, and how they relate to your participation in the program? Have you and your family talked about stroke risk factors? If so, how have you discussed them? The interview guide was revised after the first eight interviews to include follow-up questions on family support, such as: How has your family influenced your lifestyle changes? Has your family member made any lifestyle changes together with you? The interview guide was refined after the pilot study to better emphasize the family perspective and allow PWRS to describe it more clearly thoroughly. Interviews with family members include open-ended questions about: (a) expectations concerning family support, (b) experiences in changing daily habits, (c) impacts on daily life, and (d) reflections about challenges and possibilities. Examples of questions included in the interview guide: Can you describe something you did to support the PWRS during the program and the past year? Can you tell us if you noticed that the PWRS has been in the program and, if so, in what way?

## Data Analysis

Interviews (*n* = 20) were analyzed using an inductive semantic approach and reflexive thematic analysis (Braun & Clarke, 2022). Data were analyzed in the original language (Swedish). The first author KN began familiarizing themselves with the data, followed by deep immersion through reading and re-reading. In this phase, KN created reflexive notes on the content and its possible meanings, saving them in a separate document. Second, the data were carefully and systematically coded. Coding was iterative and reviewed to ensure consistency. The authors KN, EA, and LF discussed the meaning of the codes before proceeding to the next step. Third, the codes were used to form initial themes. The software program ATLAS.ti was used to support the organization of coding and notes. Throughout the third to sixth phases, all the authors KN, EA, LF, EÅ, and AHP engaged in reasoning and held several meetings to discuss the data's process and content. Two authors, KN and EA, independently translated the quotes and compared them for consistency. They discussed differences to ensure a close meaning. Between each meeting with the research group, time was allocated for individual work and reflection on the data. The study followed the Consolidated Criteria for Reporting Qualitative Research guidelines to ensure methodological rigor (Tong et al., 2007). Reflexivity was maintained throughout the research process, with the authors continuously reflecting on their assumptions, professional backgrounds, and potential influence on the data.

## Findings

A total of 20 participants were included. Twelve participants were included in dyads (six dyads), comprising a PWRS and one of their family members. Four participants participated without a corresponding dyad member. Table 1 presents the characteristics of the PWRS and family members. Table 2 describes the role of participation, relationship to the PWRS, and living situation.

**Table 1:** Characteristics of Persons at Risk for Stroke (PWRS) and Family Members (FM)

Characteristic	PWRS *N* = 10	FM *N* = 10
Age		
–Median (range)	62 (56–66)	54.5 (34–69)
Gender		
–Women	7	3
Men	6	4
Completed education		
High school	3	1
Education at a higher level	7	9
*Annually income		
$18,000—$36,000	2	2
$36,000–$55,000	4	4
< $55,000	4	4

*Median annual income in Sweden (2023): $42,876 (Statistikmyndigheten, 2023).

**Table 2:** Identification Codes, Living Situation, Dyads, and Relationship With Intervention Participants

PWRS		FM	
Identification Code	Living Situation	Identification Code	Relationship With Intervention Participant
1	With family	A	Partner
2	With family	B	Partner
3	With family	C	Partner
4	With family	D	Partner
5	Alone	E	Adult child
6	With family	F	Partner
7^a^	With family		
8^a^	With family		
9^a^	With family		
10^a^	Alone		
		G^b^	Partner
		H^b^	Adult child
		I^b^	Partner
		J^b^	Partner

Abbreviations: PWRS = persons at risk for stroke; FM = family members.

a No interview with family member.

b No interview with intervention participant.

The support that PWRS has received from their family members has been described in the interviews as verbal encouragement, confirmation, nudging, and active participation in healthy activities or in helping facilitate behavior change. Family support varies with factors such as the relationship between PWRS and family members, willingness to accept support, and expectations of support:

I have not drank as much [alcohol] when he is at home, so as not to tempt him, since he was supposed to drink less [alcohol]. (…) I have been doing this TV training (…) I have shown him such TV programs, and how he can find them (…) I have been encouraging, even though I have not gone into the details. (FM #A)

Three themes were identified: (a) sharing views about lifestyle relates to family support, (b) balancing support of lifestyle changes and respecting one's own responsibility, and (c) generating tensions from different views on health and lifestyle habits.

## Sharing Views About Lifestyle Relates to Family Support

Most family members were committed to supporting PWRS by actively participating in health-promoting activities with the PWRS and offering support and encouragement in everyday life. Explicit encouragement included asking questions, providing affirmations, and giving positive feedback on lifestyle changes. Positive feedback about the lifestyle changes encouraged PWRS and increased their motivation to continue these healthy habits. One PWRS described his partner as providing good examples and promoting health-related conversations and activities: “*Yes, I think she eats healthy, and she exercises. She is a good role model for me, so she nagged me so that she gets me on board on things” (PWRS #1).*

Family members reflected on activities and approaches that could foster support for the PWRS in making a lifestyle change. Performing health-promoting activities together that facilitate a healthier everyday life was experienced as something that contributed to adopting a lifestyle change for the PWRS. Support from the family members could include offering a ride to the gym, preparing lunch boxes for the other person, and preparing healthy meals together. Support may also involve avoiding activities, like drinking alcohol, to not tempt one's partner into unhealthy choices. One PWRS described how her partner has been supporting her lifestyle change by avoiding buying home treats and instead buying healthy products and preparing meals.

He supported me in the way that he did not buy these kinds of things [treats] for our home. (…). He brought home light products and stuff, and fish and chicken (…) that we are supposed to eat a lot of, like, or vegetarian (…) so he often cooks the food. (PWRS #3)

Family members who shared the same view of health as the PWRS expressed a stronger supportive relationship. A supportive family member who encourages and pushes the PWRS to make a lifestyle change was seen by the PWRS as an advantage. Support from family members could also enhance the PWRS's motivation to maintain the lifestyle change. A shared view on lifestyle changes, such as participating in health-promoting activities together and encouraging each other in everyday life, fosters family support and enhances participation and engagement in the lifestyle change process.

## Balancing Support of Lifestyle Changes and Respecting One's Own Responsibility

Family members expected that PWRS would take responsibility for their health and habits in everyday life to reduce risk factors for stroke. Family members often struggled to balance supporting lifestyle changes and respecting the PWRS's responsibility for their own health. Another tension described was support from family members and the PWRS inner motivation for lifestyle changes.

PWRS emphasized that it did not matter whether they received support from a family member if there was a lack of inner motivation. Both PWRS and family members stated that making lifestyle changes is a personal responsibility. Family members describe the PWRS inner motivation as coming from their own willingness to make a change. Family members faced challenges in supporting PWRS who lacked inner motivation, making it difficult to encourage them to make changes for their own sake. Family members observed a lack of inner motivation in PWRS when they did not take the initiative to, for instance, eat healthy or engage in health-promoting activities. Family members emphasized that they wanted to avoid nagging at their partner to, for example, eat healthier or exercise. However, family members wanted to support their partner. Few attempted to nudge the PWRS to make healthier choices while still hoping that the PWRS would make these lifestyle changes independently. One family member reflected on her role as a supportive wife regarding her husband's motivation and willingness to change:

I can help him by making sure that, yes, there are the right conditions around him. But then it is he himself who knows what he does and does not do. He makes his own decisions, he is an adult. (FM #D)

Family members expressed that they did not believe the PWRS had an inner motivation and could not implement a lifestyle change themselves. In these cases, family expectations about personal responsibility for lifestyle changes created tensions when family members took over certain tasks to support PWRS. For instance, family members took responsibility for grocery shopping and food preparation. One family member said that each person needs to take responsibility for their health while emphasizing that it also can be crucial to receive support in facilitating a lifestyle change. The family member shared an example of a situation from her everyday life:

We divide everything up here at home, but just with the food and this healthier stuff. He needs support with that, actually, so that it is me who prepares it. Otherwise, it is very easy for him to have a sandwich (…).If I prep him with a snack, and so he does not get this craving, then, he manages. It is actually crucial. (FM #J)

PWRS stated that having inner motivation contributed to dedication in making lifestyle changes regardless of the level of family support. They described their inner motivation as having a sense of control and confidence in the process. This sense of control encouraged them to take personal responsibility for their health. When PWRS felt motivated and successfully made lifestyle changes, their sense of control and confidence increased. One PWRS felt empowered by taking responsibility for her own lifestyle: “*I need to feel that I am the one who decides for myself. I like this autonomous kind of person who still has the power to influence my own life” (PWRS #10).*

The type of support provided by family members to the PWRS varied. Family members struggled to balance supporting the PWRS's lifestyle changes and respecting their motivations and responsibilities. This led to underlying tension, significantly when family members pushed the PWRS into healthy habits, potentially affecting their sense of control and confidence.

## Generating Tensions From Different Views on Health and Lifestyle Habits

Family members and PWRS sometimes had different views on health and lifestyle. Most family members always maintain healthy habits in their daily lives, while others have a history of sedentary behavior and unhealthy dietary habits. The variation in health behaviors may have been influenced by the support PWRS received from their families. One family member described that healthy habits have always been a natural part of her life in contradiction to her partner's habits: *“It is more important to me, but that is because it has been natural for me all my life, this thing about exercising and thinking about and eating and so on. While, what I experience he has had it” (FM #F).*

Three families held conflicting views about health and lifestyle habits. These views may arise from differing personalities and opinions on health-related questions, such as the approach to adopting a lifestyle change or varying opinions regarding what constitutes a healthy diet. One participant described a family disagreement regarding health and the motivation behind lifestyle changes. She chose not to involve her partner in her lifestyle change because her partner was focused on weight loss, while she aimed to implement changes and maintain more overall healthy habits in the long term, as she explained:

I decide for myself how it should be (…) and then he gets to do his own thing, so I have not involved him in that. In this particular case, we do not really agree, but I also understand that it is out of consideration, he is afraid that I will get too fat. (PWRS #9)

In these cases, the PWRS felt unsupported by their family members in making a lifestyle change. This support was described as varying due to the relationship between the PWRS and the family member, prioritization of other responsibilities, or lack of interest in health-related questions. Conversely, PWRS chose not to share program details with their families, preferring to pursue the lifestyle changes independently. In contrast, lack of inclusion in the program details made family members feel excluded, as they would have been more engaged if given the opportunity. Family members knew the PWRS wanted to make a lifestyle change but were uninformed about the program's content. Most PWRS did not include their family members in goal-setting or discussing activities to achieve these goals. This may be due to the focus on individual goals rather than family engagement in the MMD intervention. One family member expressed that health care generally does not consider the family as a valuable resource: “*The whole process from the healthcare system focus so much on the person who is sick that you forget to take advantage of the help and support that is available around this person” (FM #D).*

Family support could have various meanings and was sometimes entangled in contradictory messages. This ambiguity affected PWRS differently; while contradictory messages from family members often frustrated those trying to make lifestyle changes, they could also motivate some PWRS. In certain situations, family support was counterproductive due to unclear messages from family members. On one hand, family members provided explicit encouragement and engaged in behaviors promoting health-related changes. Still, on the other hand, they expressed doubt about whether it would make a difference. These conflicting messages proved counterproductive support when trying to make a lifestyle change: “*It feels good that she was involved [in the program]. It feels like she is aware, at least, a little interested (…), but, I do not really think that she will change” (FM #H).*

Four PWRS experienced ambiguous support due to contradictory messages as not credible. Despite this, PWRS were encouraged to challenge doubts about their ability to make lifestyle changes, even when family members did not believe in them. This perspective was described as empowering PWRS to take responsibility for their choices and make independent decisions about their lifestyles and habits. One PWRS used the situation as motivation to adopt new healthy habits for a lifestyle change:

I have to take control of my mind and focus on saying no, we are not going to do it [a lifestyle change] in that way, and instead say, we are going to do it this way, and in that way create a habit. (PWRS #8)

Several factors contributed to the tensions between PWRS and their family members, including differing opinions on health-related questions, ambiguity, and uncertainty about family support. Additionally, tensions might arise if PWRS felt unsupported by their family or if family members felt excluded from the lifestyle change process.

## Discussion

This study aimed to explore family support from the perspective of PWRS who participate in MMD program and their family members. The findings of this study offer insights into how family support can both facilitate and hinder lifestyle changes. Findings also provide insights into how the MMD stroke prevention program can be refined to include a family-support perspective and address how the family context influences lifestyle change. The findings align with previous research using an occupational perspective, indicating that family support can influence lifestyle change (Abel et al., 2018; Gupta et al., 2019) and that positive support from family can contribute to being able to maintain lifestyle changes (Abel et al., 2018; Mälstam et al., 2022).

### Family Support as One of the Reasons for Forward Progress

On the one hand, PWRS and their family members can shared views on lifestyle, and family members actively participated in health-promoting activities related to family support. However, on the other hand family members might found it challenging to balance offering support while respecting the PWRS's own responsibility for making lifestyle changes. In this study, family members used strategies involving nudging to encourage, rather than nag, the PWRS to adopt healthier habits change. In behavior change theory, nudging guides decisions without restricting choices or altering incentives (Thaler & Sunstein, 2022). Moreover, nudging suggests that small changes in how options are presented can encourage better decisions for individuals and society. Health-promoting behaviors within families can include members encouraging and nudging each other toward small, impactful lifestyle changes. This might include a partner buying fresh fruits instead of unhealthy snacks. This type of nudge allows PWRS to make healthier choices independently without nagging, fostering a supportive environment for healthier behaviors within the family (Matthews, 2023; Vlaev et al., 2016). Nudging can induce immediate behavioral changes, but evidence for lasting effects is limited (Ledderer et al., 2020). Although nudging can support small, meaningful behavior changes while preserving individual autonomy (Matthews, 2023), it has also been criticized for potentially undermining autonomy and dignity. The criticism argues that nudging may violate individual liberties by promoting a specific interpretation of the good that individuals might not choose for themselves (Matthews, 2023). However, creating an environment devoid of choice architecture is challenging (Thaler & Sunstein, 2022). A higher level of trust in the group providing the nudge leads to more significant approval and acceptance of the intervention (Forte et al., 2024). Trusted family members could use a nudge to support the PWRS in making decisions that align with their desire for a lifestyle change. This approach supports individuals in making better choices without losing their freedom of choice (Thaler & Sunstein, 2022). Based on the findings of this study, incorporating nudging as one strategy for family support, and theorizing how the family, as a unit, works toward the same goal, can guide professionals in PHC to target appropriate support for PWRS and family members in the MMD intervention.

### Sustainability of Lifestyle-Based Intervention Programs

Interventions, such as lifestyle programs focused on diet and physical activity at the individual level, can increase physical activity and short-term weight loss (Schneider et al., 2017; Wadden et al., 2020). However, lifestyle changes are challenging to implement and especially to maintain in everyday life (Wadden et al., 2020). The idea that making a lifestyle change is an independent endeavor is rooted in valuing personal control and confidence. However, it also raises questions about the potential lack of focus on sociality and culturally situated meanings of lifestyle change. Autonomy requires increased self-awareness of the personal, cultural, social, and environmental factors that impact behavior (Bates et al., 2024). Based on the findings of this study, family support, which can potentially disrupt individual autonomy, can enhance motivation and, consequently, facilitate lifestyle change. Within the framework of behavior change theory (Michie et al., 2013), family support can enhance both social opportunity and motivation, boosting the likelihood of lasting behavior change. By providing emotional encouragement and practical help, family members foster an environment where individuals feel supported in adopting and sustaining healthy habits behaviors. Bandura (2006) contends that self-efficacy is the key mechanism of human agency (Bandura, 2006). It manifests through both individual and family-level factors, driving changes in health behaviors and aiding in achieving health-related goals (Ho et al., 2022). However, occupational scientists Fritz and Cutchin (2017) have also raised critiques of current health behavior theories, which can lead to a lack of maintained behavioral change over time. From a transactional perspective, the individual and the environment are viewed as a dynamic unit in which actions and situations influence one another. Integrating perspectives about how individuals and their environments, including family support, interact can make interventions more effective and sustainable over time (Fritz & Cutchin, 2017). Previous studies have also reported the importance of social contexts in maintaining healthy habits from an occupational perspective (Mälstam et al., 2022; Moll et al., 2015; Reitz & Scaffa, 2020).

Finally, the different views on health and lifestyle habits theme created tensions, shaped expectations, and raised questions about roles and the varying expectations regarding family support. During data collection, neither the PWRS nor family members explicitly mentioned gender differences in actions related to family support. However, the findings revealed how gendered understandings of family support can be expressed in everyday situations of meal preparation, nudges, and in doing or being together.

This distinction highlights gendered expectations within roles, indicating that gender stereotypes can influence family perceptions about support and agency in lifestyle changes at home (Heise et al., 2019; Nyman et al., 2018)

### Methodological Considerations

A strength of this study is its inclusion of two perspectives on family support: family members and intervention participants (PWRS). The interaction between PWRS and family members offers insights into family roles and support dynamics. This approach deepens the understanding of when support is beneficial and when challenges arise. Another limitation was the homogeneity of the PWRS and family groups, which were mainly composed of highly educated individuals born in Sweden. Research indicates that patients and families often share similar concerns, regardless of ethnicity (Astin et al., 2008). However, using reflexive thematic analysis (Braun & Clarke, 2022) allowed for capturing nuanced perceptions and identifying patterns within both groups. Reflective analysis meetings among the authors enhanced reflexivity, ensuring a more comprehensive understanding of family support among PWRS and their families.

A limitation of this study is that social bias could have affected data collection. PWRS were interviewed face-to-face, while family members were contacted by phone, possibly influencing response depth and comparability. In-person interviews are more time-consuming but enable observation of body language and facial cues. In contrast, telephone interviews are quicker and can include multiple family members who might not be able to participate because of time or travel limitations (Saarijarvi & Bratt, 2021). Considering the purpose of this study, the research team agreed that using both methods was suitable and would not impact the interview content (Opdenakker, 2006). Finally, revisions to the interview guide after the first eight interviews could have affected data collection, leading to differences in data depth. Additionally, limited demographic diversity restricts the transferability of the findings.

## Conclusion

This study explored family support from the perspective of PWRS who participate in MMD intervention and their family members. The findings highlight perspectives on family support from PWRS and family members, including the complexities involved in providing and receiving support. Family support depends on the relationship between PWRS and their families, the willingness to accept support, and expectations of support. Lifestyle change can be facilitated when a committed family member participates in health-promoting activities with PWRS or explicitly encourages the lifestyle change by giving positive feedback. The findings also reveal tensions related to family support, especially when family members face challenges balancing support for lifestyle changes while respecting the PWRS own responsibility. Despite these tensions, incorporating social context, including family support, into lifestyle programs can facilitate lifestyle change and enhance the sustainability of occupation-based lifestyle interventions.

## Key Messages

- Shared participation in health-promoting activities and a shared understanding of health and lifestyle within the family were essential to supporting the PWRS in adopting and maintaining healthy lifestyle changes.
- Understanding how the PWRS interacts with their environment, including family support, can enhance the sustainability of occupation-based lifestyle interventions.
- Using nudging as a family-based support strategy in the MMD intervention could promote sustainable lifestyle changes for PWRS and their families.
